# Metabolomics Reveals the Mechanism by Which Sodium Butyrate Promotes the Liver Pentose Phosphate Pathway and Fatty Acid Synthesis in Lactating Goats

**DOI:** 10.3390/ani14223249

**Published:** 2024-11-13

**Authors:** Lin Li, Xi Chen, Shuping Yan, Yuanshu Zhang

**Affiliations:** 1 School of Chemical Engineering and Biotechnology, Xingtai University, Xingtai 054001, China; linl1991@163.com; 2 Key Laboratory of Animal Physiology and Biochemistry, Ministry of Agriculture, Nanjing Agricultural University, Nanjing 210095, China; hlliu1960@126.com (X.C.); 201911223@xttc.edu.cn (S.Y.); 3 Hebei Key Laboratory of Digital Freshwater Aquaculture Technology, Xingtai University, Xingtai 054001, China

**Keywords:** ruminants, subacute rumen acidosis, butyric acid, liver metabolism, milk fat

## Abstract

This study aimed to explore the effects of sodium butyrate on liver metabolism in goats subjected to a high-concentrate diet. We randomly assigned twelve Saanen-lactating goats into two groups, one of which received a high-concentrate diet (concentrate: forage = 60:40, control group), while the other received the same basal diet supplemented with sodium butyrate (SB) (10 g/kg basal diet, SB group). Compared with the control diet, the SB diet considerably increased the milk fat percentage and content (*p* < 0.05), with an increase of 0.67% in the milk fat content of the SB group. By employing a global metabolomics approach based on ultra-performance liquid chromatography–tandem mass spectrometry (UPLC–MS/MS), we identified 6748 ions in ESI+ mode and 3573 ions in ESI− mode after liver isolation from both groups. A total of twenty-three metabolites, including phospholipids, fatty acids, and ribose phosphate, were found to be dysregulated according to a search against the human metabolome database (HMDB). Pathway analysis revealed activation of the pentose phosphate pathway, glycerophospholipid metabolism, and unsaturated fatty acid synthesis. The SB diet also modulated the expression of key lipogenic enzymes, such as acetyl-CoA carboxylase (ACC) and stearoyl-CoA desaturase (SCD-1), which are downstream targets of the transcription factor sterol regulatory element-binding proteins-1c (SREBP-1c), inducing a significant upregulation (*p* < 0.05). Furthermore, 6-phosphogluconate dehydrogenase (6PGDH) levels in the liver were elevated after the lactating goats were fed the SB diet (*p* < 0.05). Our study reveals that the SB diet may offer substantial benefits in enhancing the milk quality of subacute ruminal acidosis (SARA) goats. This is accomplished by augmenting the activity of the liver pentose phosphate pathway and the process of de novo fatty acid synthesis in lactating goats.

## Simple Summary

Long-term consumption of high-concentrate diets leads to subacute ruminal acidosis (SARA). Butyric acid is a small molecule that can inhibit the growth of harmful bacteria through cell membranes. As a type of organic acid, butyric acid functions to regulate body material metabolism and improve body immunity. Our findings suggest that a high-concentrate diet with sodium butyrate (SB) improves the SARA status of lactating goats. In addition, metabolomics studies revealed that an SB diet can activate the liver pentose phosphate and lipid anabolism pathway, causing changes in fatty acid distribution and redistribution in the liver. Subsequently, more milk fat precursors (fatty acids) enter the mammary gland through the milk artery to synthesise milk fat.

## 1. Introduction

Ruminants are commonly supplied with a diet rich in concentrated nutrients to fulfil the nutritional requirements for high milk production during lactation [1]. However, the prolonged ingestion of high-concentrate (HC) diets can precipitate subacute ruminal acidosis (SARA). SARA, a prevalent digestive ailment, is characterised by a persistently low rumen pH level, falling below 5.8, for at least 5 h post-feeding each day [2]. As an immune organ, the liver plays a role in processing, digestion and absorption, protein synthesis, and detoxification [3]. Excessive lipopolysaccharide accumulation in the liver causes damage and decreases in defence functions, leading to inflammation [4]. Studies have shown that high-concentrate feeding can affect the genes involved in lipid metabolism in the liver [5]. A reduction in the delivery of free fatty acids from the liver to the bloodstream correlates with diminished milk fat synthesis, as these acids are integral to the process [6]. Therefore, milk fat synthesis is negatively impacted by a high-concentrate diet, which disrupts liver lipid anabolism. Furthermore, our previous research showed that in ruminants, after being fed a HC diet, the expression profiles of genes related to the inflammatory response and lipid metabolism in the liver changed significantly [7].

Butyric acid is a small molecule that can inhibit the growth of harmful bacteria through cell membranes [8]. It can also promote the growth of beneficial bacteria in the gut for a better balance in the gastrointestinal flora [9]. The addition of sodium butyrate to feed can increase animal food intake and animal weight [10]. In addition, after sodium butyrate is digested and absorbed in the intestinal cavity, it can provide energy for the intestinal epithelial cells through oxidation and play a role in repairing the mucosal epithelial cells [11]. Furthermore, sodium butyrate is known to regulate metabolic processes within the body and enhance immune function. With this in mind, we introduced sodium butyrate into goats experiencing SARA to explore its effect on the liver metabolic expression profile.

Metabolomics refers to the thorough examination of all the metabolites present in the cells, tissues, or biofluids in terms of quantity and quality. In a recent study, Zhang et al. used metabolomics and proteomics to explore liver metabolism in transition cows, revealing the upregulation of gluconeogenesis, fatty acid oxidation, and other energy utilisation pathways postpartum [12]. Ghaffari et al. used metabolomics to investigate the metabolic changes in the livers of dairy cows from dry-off to calving [13]. Despite advancements in the understanding of the metabolic changes during peripartum and early lactation, gaps remain, particularly with respect to liver metabolism and its influence on milk quality. This study employed metabolomic technologies to investigate the precise impact of sodium butyrate on liver metabolites in lactating goats experiencing SARA. Our goal was to deepen our understanding of the interplay between liver metabolism and milk quality, with the ultimate aim of identifying a possible method to improve dairy quality.

## 2. Materials and Methods

### 2.1. Animals and Experimental Procedures

Twelve healthy multiparous mid-lactating Saanen dairy goats were acquired from the Northwest A&F University test farm. They were housed in individual cages at the Experimental Animal Centre (Nanjing Agricultural University, China). The goats had similar weights and lactation periods (39 ± 7 kg (mean ± SEM kg), 2–3 weeks postpartum). After one week of adaptation to the high-concentrate feed, all the animals were fitted with rumen fistulas, and the experimental animals were allowed to recover for two weeks after the operation. Six lactating goats per group were assigned to each of the high-concentration (Control) group or sodium butyrate (SB) group following the recovery period. Both groups of goats received identical basal diets with a concentrate/forage ratio of 60:40. Table 1 displays the chemical composition of the basal diet. The SB group was fed a basic diet supplemented with sodium butyrate (10 g/kg basal diet; Sigma, Saint Louis, MO, USA). Throughout the 20-week duration of the experiment, the animals were fed in their cages and given free access to water. A total of 1 kg of basal diet (dry matter) was offered to the goats twice daily (at 08:00 and 18:00) while they were milked. We also documented the amount of milk produced each day. To determine the milk composition, the samples were analysed via an Integrated Milk-Testing™ Milkoscan 4000 (Foss Electric, Hillerod, Denmark) at the Weigang Dairy Testing Center in Nanjing, Jiangsu Province, during the 20th week.

### 2.2. Rumen Fluid Collection and Analysis

At week 20 of the experiment, before feeding in the morning (0 h), ruminal fluid was collected from the ventral sac of the rumen through the rumen fistula. It was then collected at 2, 4, 6, 8, and 10 h after feeding. After the rumen fluid was filtered with a 4-layer gauze, the pH of the rumen fluid was assessed with a pH meter (HI 9125, Hanna Instruments, Woonsocket, RI, USA).

### 2.3. Sample Collection

At the end of the experiment, each goat was anaesthetised with 0.5 mL of xylazine hydrochloride. All the goats were slaughtered after blood samples were collected, and liver tissue was collected and divided into small pieces. Liquid nitrogen was used to chill the livers before RNA and protein extraction.

### 2.4. Liver Metabolite Extraction

The livers of the goats were quickly removed, immersed in liquid nitrogen, and then preserved at −80 °C so that the tissue metabolome could remain as intact as possible until processing. The 25 mg liver samples were milled for 5 min after mixing with 800 μL of a 1:1 ratio of precooled methanol/water mixture. The mixture was centrifuged at 30,000× *g* for 20 min at 4 °C in a centrifuge. The 550 μL liquid phase was then vacuum-frozen and transferred to a fresh 2 mL polypropylene tube for liquid chromatography separation.

### 2.5. UPLC–MS/MS Analysis

An ACQUITY UPLC HSS T3 column manufactured by Waters in the United Kingdom was used throughout the separation process. The column dimensions were 100 mm by 2.1 mm, with particles that were 1.8 μm in size. At a flow rate of 0.4 mL/min, the temperature of the column was maintained at a constant 50 °C.

Two phases were used for the binary mobile phase system, namely mobile phase A and mobile phase B. A gradient elution approach was used to separate the chemicals via chromatography, with water and 0.1% formic acid as mobile phase A and 0.1% methanol and formic acid as mobile phase B. The proposed precise sequence of steps was 0 to 2 min at 100% A, from 2 to 11 min at 0% to 100% B, from 11 to 13 min at 100% B, and finally, from 13 to 15 min at 0% to 100% A. The flow rate was adjusted to 0.4 mL per min, and the syringe volume was set at 10 µL.

A high-resolution tandem mass spectrometer, the Xevo G2 XS QTOF, manufactured by Waters in the United Kingdom, was used to identify the metabolites discharged from the column. The Q-TOF was used when working with both positive and negative ions. The voltage of the sample cone was set to 40.0 V while the device was operating in positive ion mode, and the voltage of the capillary was set to 3.0 kV. The capillary voltage was 2.0 kV, and the sample cone voltage was 40.0 V. The centroid MSE mode was used to produce data from the mass spectrometer. Within a fraction of a second, we traversed the TOF mass band spanning from 50 to 1200 Da. We employed a scan period of 0.2 s and 20–40 electron volts for the mass spectrometry (MS/MS) experiments to break up the precursors. To ensure that the mass was accurate, the LE signal was captured at regular intervals of three seconds during the collection operation. After each set of ten samples, a quality control sample, which was a pool of all samples, was obtained to determine the degree to which the LC–MS data remained stable throughout the collection process.

### 2.6. Data Processing

Given that more than 50% of the QC samples are absent, the LOESS signals were corrected by applying QC-RSC, which relies on QC sample data, to the actual sample signals. Data were discarded if more than 80% of the ions were absent from the actual sample or if all the QC samples had a relative standard deviation (RSD) greater than 30%. Thus, ions exhibiting an RSD greater than 30% were not included in further statistical analysis because of their high degree of experimental fluctuation. Principal component analysis (PCA) revealed that a small number of components best represented the original data. Log2 conversion, scaling, and Pareto scaling were applied to the data in this project before the principal component analysis (PCA) [12]. This study sought to identify the metabolites with differential expression by combining a univariate analysis of the fold-change and q values with VIP values from a multivariate PLS-DA model’s first two main components. The selection criteria were as follows: When three requirements were met—(1) VIP ≥ 1, (2) fold change ≥1.2 or ≤0.8333, and (3) q value < 0.05—the differential ion, also known as the common ion, was generated. The KEGG database was used to analyse the metabolic pathways. The data were transformed via a log2 function to prepare them for cluster analysis. The cluster analysis was performed with the pheatmap tool in R.

### 2.7. RNA Isolation, cDNA Synthesis, and Real-Time PCR

Total RNA was immediately extracted from the appropriate liver tissues via TRIzol [14]. Afterwards, the concentration and purity of the total extracted RNA were determined via a NanoDrop 2000 (Thermo Scientific, Waltham, MA, USA). The purity of total RNA was determined by analysing the OD260/OD280 values. The exact operational procedure was carried out according to the directions given by the Vazyme Company to generate cDNA from 1 μg of total RNA via reverse transcription. The primer sequences are shown in Table 2. Fluorescence quantitative PCR and the 2^−∆∆Ct^ method were used to determine the relative amount of mRNA expression. The qPCR mixture had a total volume of 20 μL, including 2 μL of primer, 10 μL of SYBR Green, 6 μL of ddH_2_O, and 2 μL of cDNA. An internal standard, the GAPDH butyl gene, was used. The annealing temperature and primer concentration were adjusted. The PCR settings were as follows: 40 cycles of 2 min of predenaturation at 95 °C, 15 s of denaturation, and 45 s of annealing at 60 °C. The specificity of the PCR products was verified by melting curve analysis after PCR. Three runs were performed on each tissue sample.

### 2.8. Western Blot Analysis

After the samples were frozen, the total protein level was evaluated via a bicinchoninic acid (BCA) assay kit (Pierce, Rockford, IL, USA). For each sample, 30 µg of total protein was subjected to 10% SDS-PAGE. Protein purification was then performed, which included loading the proteins onto nitrocellulose membranes manufactured by BioTrace and provided by Pall Co., New York, NY, USA. The blots were incubated overnight at 4 °C, at dilutions of 1:1000 in the blocking solution, and the main antibodies used were anti-6-phosphogluconate dehydrogenase (rb-anti-6PGDH, #13389, CST), and anti-glucose 6-phosphate dehydrogenase (rb-anti-G6PDH, #12263, CST). First, the blots were treated with a primary antibody against GAPDH (A531, Bioworld, Nanjing, China; 1:10,000) as an extra normalisation layer. The membranes were washed and incubated at room temperature for 2 h with a secondary antibody conjugated with HRP. Enhanced chemiluminescence (ECL) was used to detect the signal after the blots were washed with LumiGlo substrate (Super Signal West Pico Trial Kit, Pierce, MO, USA). We used Quantity One software (https://www.bio-rad.com/en-cn/product/quantity-one-1-d-analysis-software?ID=1de9eb3a-1eb5-4edb-82d2-68b91bf360fb, accessed on 10 November 2024), created by Bio-Rad (Hercules, CA, USA), to import the ECL signal from the imaging equipment.

### 2.9. Statistical Analysis

The results are presented as the means ± SEMs. The ruminal pH data for the detection of differences due to diet and feeding time between these variables were analysed via univariate analysis via general linear models in SPSS 20.0 for Windows (StatSoft, Inc., Tulsa, OK, USA). All other data were analysed via an independent sample *t* test and tested for statistical significance to validate the normality of the data. Results were considered statistically significant when the *p* value was less than 0.05.

## 3. Results

### 3.1. Effect of Sodium Butyrate on the Milk Composition of Lactating Goats Fed a High-Concentrate Diet

At the 20th week, treatment with sodium butyrate did not affect milk yield (*p* = 0.136) or milk protein content (%, *p* = 0.086; g/d, *p* = 0.479). The SB diet led to a notable increase in the fat percentage and content in the milk (*p* < 0.05, Table 3).

### 3.2. Rumen pH in the Rumen

**Figure 1:**
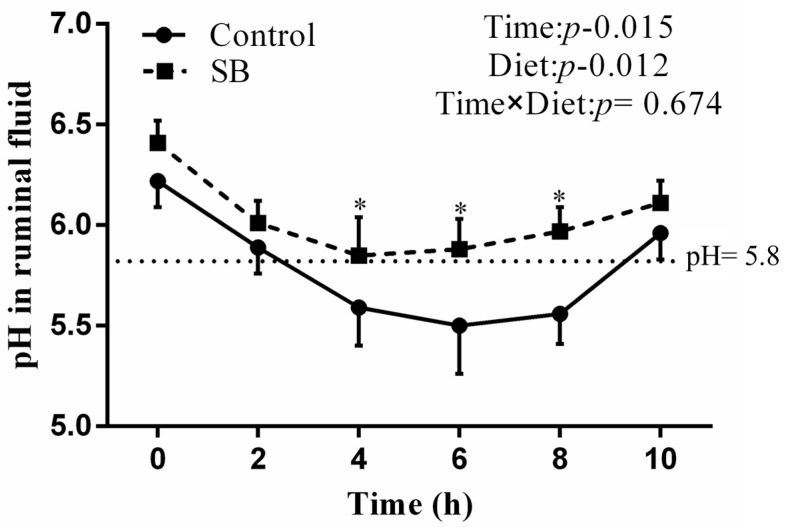
pH values of ruminal fluid after a 20-week feeding regimen. The data are presented as the means ± SEMs (*n* = 6/group). Data were analysed for differences due to diet, time, and variable interactions via univariate analysis via general linear models in SPSS 20.0 for Windows. * *p* < 0.05 indicates a statistically significant difference compared with the control group.

After 20 weeks of SB diet consumption, the rumen pH profile of the SB goats was higher than that of the control goats. The results revealed that a pH under 5.8 lasted 5 h in the control goats, indicating that SARA was successfully induced. Compared with the control goats, the SB goats presented significantly higher pH values (*p* < 0.05, *p* = 0.012). The digestion time significantly affected the ruminal pH, whereas there was no interaction effect between the digestion time and the diet or ruminal pH (Figure 1).

### 3.3. Analysis of the Liver Metabolism Patterns in Lactating Goats Fed a HC Diet with Sodium Butyrate

**Figure 2:**
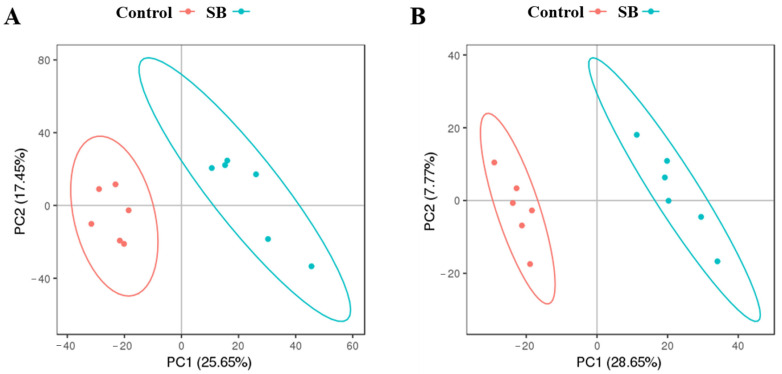
PLS-DA score plot of control and SB goats. (**A**) The positive ion mode score plot has an R^2^ of 0.9581 and a Q^2^ of 0.7014; (**B**) The negative ion mode score plot has an R^2^ of 0.9878 and a Q^2^ of 0.8175. The distance between points in the score plot shows how comparable the samples are, with each point representing a goat liver sample. The first and second axes represent PC1 and PC2, respectively.

In each sample profile, 6748 ions were detected in positive (POS) mode, and 3573 were detected in negative (NEG) mode. During the study, six QC samples were examined in addition to the goat liver samples. The LC–MS–MS/MS system demonstrated remarkable stability and consistency, as evidenced by the tight clustering of the QC samples in the PCA (Figure 2). After the exclusion of ions with an RSD greater than 30%, 5050 ions were detected in each POS mode sample (Figure 2A), and 2751 ions were detected in each NEG mode sample (Figure 2B).

### 3.4. Screening of Differentially Abundant Metabolites in the Liver

**Figure 3:**
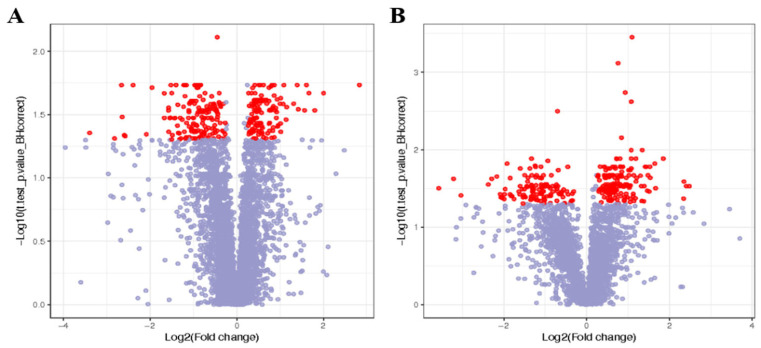
Metabolite volcano plot. The red dots represent differentiated metabolites, and the purple dots represent undifferentiated metabolites. (**A**) PCA of positive ion mode; (**B**) PCA of negative ion mode.

This investigation included goat liver samples and six QC samples. Principal component analysis (PCA) revealed that the QC samples were closely grouped, highlighting the exceptional consistency and stability of the LC–MS–MS–MS/MS technology (Figure 3). After ion samples with an RSD greater than 30% were removed, 5050 ions in POS mode samples (Figure 3A) and 2751 ions in NEG mode samples were discovered (Figure 3B).

### 3.5. Pathway Analysis of Differentially Abundant Liver Metabolites

**Figure 4:**
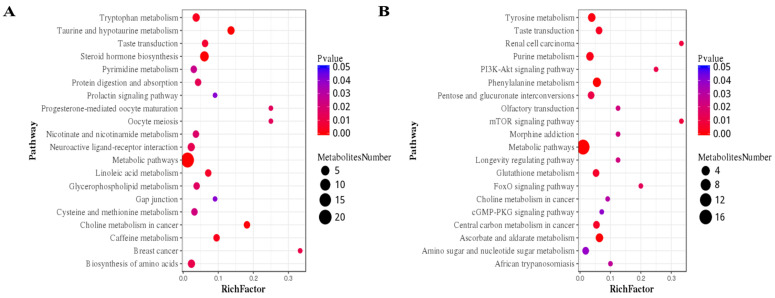
Metabolite set enrichment analysis of liver differentially abundant metabolites in goats. (**A**) KEGG pathway enrichment analysis of metabolites detected in positive ion mode; (**B**) KEGG pathway enrichment analysis of metabolites detected in negative ion mode.

The influence of sodium butyrate on various metabolic pathways in lactating goats fed a high-concentrate diet was investigated through enrichment analysis. As shown in Figure 4, 44 unique metabolites in POS mode were associated with 20 different pathways, including metabolic routes, the production of steroid hormones, taurine and hypotaurine metabolism, and tryptophan metabolism (Figure 4A). In NEG mode, 37 unique metabolites were linked to 20 metabolic pathways, including tyrosine, purine, and phenylalanine metabolism (Figure 4B).

A total of 23 ion masses, including positive and negative mode ions, were discovered via a database search of the mass-based human metabolome database (HMDB). These findings are preliminary. Table 4 lists the assumed metabolites for the positive (POS) and negative (NEG) modes. In the POS mode, the expression of metabolites related to the pentose phosphate pathway, fatty acid biosynthesis, linoleic acid metabolism, and unsaturated fatty acid biosynthesis increased. In the NEG mode, metabolites associated with the pentose phosphate pathway and the production of unsaturated fatty acids were upregulated. Sodium butyrate is a potential tool for improving the pentose phosphate pathway and lipid synthesis in goat livers.

### 3.6. Sodium Butyrate Treatment Regulated Key Enzymes Involved in Lipid Metabolism in the Livers of Goats

**Figure 5:**
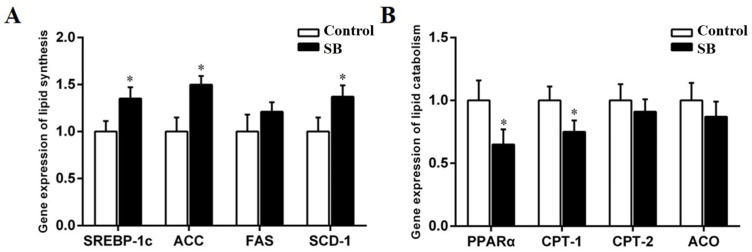
Effects of sodium butyrate on the expression of liver lipid metabolism in lactating goats. (**A**) Genes involved in lipid synthesis, sterol regulatory element-binding protein-1c (SREBP-1c), acetyl-CoA carboxylase (ACC), fatty acid synthetase (FAS), and stearoyl-CoA desaturase 1 (SCD-1), were measured in liver tissue. (**B**) Genes involved in lipid catabolism, peroxisome proliferator-activated receptor α (PPARα), carnitine palmitoyl transferase-1 (CPT-1), carnitine palmitoyl transferase-2 (CPT-2), and acyl-CoA oxidase (ACO), were measured in liver tissue. The data are presented as the means ± SEMs (*n* = 6/group). * *p* < 0.05 indicates a statistically significant difference compared with the control group.

The expression of SREBP-1c was considerably higher in the livers of the SB goats (*p* < 0.05). The SB diet also increased the expression of SREBP-1c downstream targets, which included ACC, FAS, and SCD-1. The expression of ACC and SCD-1 was noticeably higher in the SB group than in the control group, as shown by *p* < 0.05 (ACC, *p* = 0.015; SCD-1, *p* = 0.012; Figure 5A). Compared with the control group, the SB group presented significantly lower PPARα and CPT-1 expression levels (*p* < 0.05; Figure 5B). In contrast, CPT-2 and ACO expression did not differ significantly between the SB and control groups (CPT-2, *p* = 0.258; ACO, *p* = 0.157).

### 3.7. Sodium Butyrate Treatment Regulated Key Enzymes of the Pentose Phosphate Pathway in the Livers of Goats

**Figure 6:**
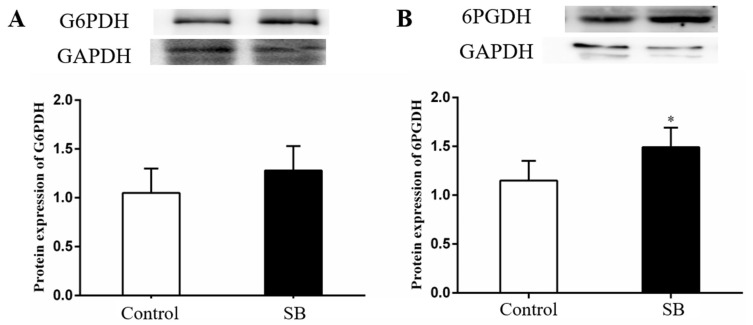
Effects of sodium butyrate on the expression of the G6PDH and 6PGDH proteins in the livers of lactating goats. Pentose phosphate pathway genes involved in glucose-6-phosphate dehydrogenase (G6PDH) and 6-phosphogluconate dehydrogenase (6PGDH) were measured in liver tissue. The data are presented as the means ± SEMs (*n* = 6/group). * *p* < 0.05 indicates a statistically significant difference compared with the control group. (**A**) Western blot analysis of G6PDH protein expression in the liver affer treatment with an SB diet; (**B**) Western blot analysis of 6PGDH protein expression in the liver affer treatment with an SB diet.

Figure 6 shows that the SB group presented a higher liver expression of the G6PDH (*p* = 0.272) and 6PGDH (*p* = 0.032) proteins than the control group. In particular, there was a significant increase in the 6PGDH expression compared with that of the control group (*p* < 0.05).

## 4. Discussion

At present, dairy goats are frequently provided with HC diets to satisfy their energy requirements for achieving high milk production. Nonetheless, the consumption of such HC diets poses a significant risk to the health of these animals. It is widely documented that the feeding of HC diets to ruminants can lead to SARA, a prevalent metabolic disorder that frequently affects animals with high production levels [15]. The pH of rumen fluid is a critical determinant of dietary fermentation, serving as a key marker of the balance between the synthesis and elimination of short-chain fatty acids in the rumen [16]. Adequate fermentation in the rumen is only possible by maintaining the pH within a range that is considered natural (5.8 to −7.0). There is a general consensus that a pH below 5.8 that is sustained for over four hours is indicative of SARA. In the present study, goats fed a high-concentrate diet for 20 weeks presented with a lower ruminal pH, and the pH increased but remained stable below 5.8 for more than 5 h per day after feeding. Additionally, according to the definition of the experimental SARA, the control group had SARA.

The active ingredient of sodium butyrate is butyric acid, which is produced by the gut flora through the fermentation of nonabsorbable carbohydrates and proteins, such as fibre [17]. As an additive in animal feed, sodium butyrate is recognised for improving animal growth performance, reducing the risk of mastitis, and increasing the milk fat content [18,19,20]. Research has shown that long-term consumption of a high-concentrate diet can lead to metabolic disorders in goats, resulting in a decrease in milk fat content [21]. It has previously been reported that the addition of sodium butyrate to diets can regulate metabolism and improve lactation performance [22,23]. Our results revealed that the fat percentage and fat content of the SB goats were higher than those of the control goats. Sodium butyrate is anticipated to improve milk quality, particularly in animals affected by SARA. However, the mechanism by which sodium butyrate regulates milk fat still requires further study.

Milk fat, a vital nutritional component of milk, is known for its health benefits. However, an HC diet over an extended period can lead to a decrease in milk fat [24]. Triglycerides, the primary constituents of milk fat, are synthesised in mammary epithelial cells from fatty acids and α-glycerophosphate. The mammary glands’ absorption of nonesterified fatty acid (NEFA) components is influenced by their blood concentrations. Research has demonstrated that as the NEFA levels in the blood rise, so does the amount absorbed by mammary cells for the synthesis of milk fat [25]. As a result, NEFAs play a pivotal role as substrate precursors in the process of milk fat synthesis. The nutrients essential for milk synthesis must journey from the rumen and intestine to the liver, where they undergo metabolic transformation. The liver plays a pivotal role in animals, serving as a key regulatory organ for lipid metabolism and ensuring the maintenance of lipid homeostasis. In ruminants, the liver plays a pivotal role as the central hub for digestion and lipid metabolism, supplying the necessary substrate precursors to the mammary gland for efficient milk production [26].

We explored the effects of sodium butyrate on metabolic changes in the liver through metabolomics techniques. Our metabolomics data highlighted the differential expression of key metabolites between the two groups. Notably, the present study revealed an increase in specific fatty acids, such as eicosadienoic acid, xylulose 5-phosphate, ribose 1-phosphate, docosapentaenoic acid, and d-ribose 5-phosphate, in POS mode. In addition, there was an increased presence of eicosapentaenoic acid and 6-phosphogluconic acid in the NEG mode. These metabolites, which are linked to unsaturated fatty acid biosynthesis and the pentose phosphate pathway according to KEGG database analysis, suggest a regulatory role of sodium butyrate in the liver metabolism of lactating goats.

Further investigations into the hepatic levels of crucial enzymes and transcription factors that regulate lipid metabolism were conducted to understand the mechanism by which SB treatment stimulates lipogenesis. Sterol regulatory element-binding proteins (SREBPs) act as transcription factors for lipid and fatty acid synthesis [27]. SREBP-1c, a family member, regulates the expression of ACC, SCD-1, FAS, and the other genes involved in the biosynthesis and deposition of lipids [28]. PPARα plays an important role in regulating mitochondrial and peroxisomal fatty acid oxidation in ruminants, including the modulation of downstream targets, such as L-FABP, CPT-1, CPT-2, and ACO [29,30]. Previous studies have also shown the regulation of hepatic genes related to lipid metabolism by sodium butyrate supplementation in rats [31]. Our study demonstrated a significant upregulation of SREBP-1c in the SB group, with the expression of the downstream genes ACC and SCD-1 reflecting this increase. In contrast, the mRNA levels of PPARα and its target CPT-1 were found to be decreased. These findings indicate that liver synthesis of new fatty acids may be increased in goats subjected to SB treatment.

The pentose phosphate pathway (PPP) is essential for the liver [32]. The oxidative PPP generates two nicotinamide dinucleotide hydrogen phosphate (NADPH) and ribose-5-phosphate (R5P) molecules through the enzymes glucose-6-phosphate dehydrogenase (G6PDH) and 6-phosphogluconate dehydrogenase (6PGDH) [33]. NADPH, produced by this pathway, is crucial for lipid synthesis and redox homeostasis [34]. G6PDH, the rate-determining enzyme of the first step of the pentose phosphate pathway, converts glucose 6-phosphate (G6P) into 6-phosphogluconolactone. Thus, this pathway produces NADPH, which is essential for reductive biosynthesis and cellular oxidation–reduction reactions. Previous studies have also shown that the intraperitoneal injection of a certain dose of sodium butyrate into mice can activate the pentose phosphate pathway in brain tissue [35]. In our study, the 6PGDH proteins in the liver were highly expressed after SB treatment, suggesting that the SB diet may promote the pentose phosphate pathway in the livers of goats. The activation of the PPP enhances the hepatic synthesis of fatty acids. These fatty acids are then released from the liver via the hepatic vein, subsequently entering the bloodstream where they are absorbed. The fatty acids are then further transported into the mammary gland via the milk arteries, where they are utilised for the synthesis of milk fat.

## 5. Conclusions

Our research indicates that a diet supplemented with sodium butyrate (SB diet) improves the SARA status of lactating goats. Metabolomic studies showed that the activation of the liver pentose phosphate and lipid anabolism pathways by the SB diet leads to the altered distribution and redistribution of fatty acids within the liver. Consequently, more milk fat precursors (fatty acids) can enter the mammary gland through the milk artery to synthesise milk fat.

## Figures and Tables

**Table 1:** Composition and nutrient levels of basal diets.

Concentrate: Forage Ratio 60:40			
Ingredient (% of Dry Matter)		Nutrient Level ^b^	
*Leymus chinensis*	27.00	Net energy/(MJ·kg^−1^)	6.71
Alfalfa silage	13.00	Crude protein/%	16.92
Corn	23.24	Neutral detergent fibre/%	31.45
Wheat bran	20.77	Acid detergent fibre/%	17.56
Soybean meal	13.67	Calcium/%	0.89
Limestone	1.42	Phosphorus/%	0.46
NaCl	0.30		
Premix ^a^	0.60		
Total	100.00		

^a^ Guaranteed levels provided per kg of diet: vitamin A: 6.000 IU/kg (min); vitamin D: 2.500 IU/kg (min); vitamin E: 20.000 IU/kg (min); Cu: 6.25 mg/kg (min); Fe: 62.5 mg/kg (min); Zn: 62.5 mg/kg (min); Mn: 50 mg/kg (min); I: 0.125 mg/kg (min); Co: 0.125 mg/kg (min). ^b^ Nutrient levels were estimated values.

**Table 2:** Primer sequences used for qRT-PCR analysis of target genes in lactating goats.

Target Genes	Primer Sequences (5′-3′)	Products/bp
GAPDH	GGGTCATCATCTCTGCACCT GGTCATAAGTCCCTCCACGA	177
ACC	ACGCAGGCATCAGAAGATTA GAGGGTTCAGTTCCAGAAAGTA	179
FAS	GCACTACCACAACCCAAACCC CGTTGGAGCCACCGAAGC	161
SCD-1	CCGCCCTGAAATGAGAGATG AGGGCTCCCAAGTGTAACAGAC	154
SREBP-1c	CGACTACATCCGCTTCCTTCA ACTTCCACCGCTGCTACTG	259
CPT-1	CCCATGTCCTTGTAATGAGCCAG AGACTTCGCTGAGCAGTGCCA	230
CPT-2	ACGCCGTGAAGTATAACCCT CCAAAAATCGCTTGTCCCTT	119
ACO	TAAGCCTTTGCCAGGTATT ATGGTCCCGTAGGTCAG	189
PPARα	GGAGGTCCGCATCTTCCACT GCAGCAAATGATAGCAGCCACA	352

GAPDH: glyceraldehyde 3-phosphate dehydrogenase; ACC: acetyl-CoA carboxylase; FAS: fatty acid synthetase; SCD-1: stearoyl-CoA desaturase 1; SREBP-1c: sterol regulatory element-binding protein-1c; CPT-1: carnitine palmitoyl transferase-1; CPT-2: carnitine palmitoyl transferase-2; ACO: acyl-CoA oxidase; PPARα: peroxisome proliferator-activated receptor α.

**Table 3:** Effect of sodium butyrate on the performance of lactating goats.

	Control	SB	*p* Value
Milk yield, g/d	988.25 ± 31.34	1024.14 ± 26.18	0.136
Milk protein			
%	2.82 ± 0.11	3.04 ± 0.02	0.086
g/d	29.35 ± 1.24	30.04 ± 0.35	0.479
Milk fat			
%	3.02 ± 0.07	3.69 ± 0.14 *	0.015
g/d	34.46 ± 0.23	37.35 ± 0.73 *	0.048

The data are presented as the means ± SEMs (*n* = 6/group). * *p* < 0.05 indicates a statistically significant difference compared with the control group.

**Table 4:** List of differentially abundant metabolites identified in POS and NEG modes.

Mode	Metabolites	FC	*p* Value	VIP	Trend	Metabolic Pathways
POS	Bovinic acid	1.45	0.01	1.61	up	Linoleic acid metabolism
	Linoleic acid	1.45	0.01	1.61	up	Linoleic acid metabolism
	Palmitoleic acid	3.04	0.01	2.77	up	Fatty acid biosynthesis
	Dodecanoic acid	0.59	0.03	1.81	down	Fatty acid biosynthesis
	Eicosadienoic acid	1.60	0.02	1.66	up	Biosynthesis of unsaturated fatty acids
	Docosapentaenoic acid	1.37	0.03	1.46	up	Biosynthesis of unsaturated fatty acids
	Glycerophosphocholine	1.25	0.04	1.14	up	Glycerophospholipid metabolism
	Phosphorylcholine	1.36	0.04	1.26	up	Glycerophospholipid metabolism
	LysoPC (18:3 (6*Z*, 9*Z*, 12*Z*))	0.71	0.02	1.35	up	Glycerophospholipid metabolism
	LysoPC (22:4 (7*Z*, 10*Z*, 13*Z*, 16*Z*))	0.52	0.04	2.08	down	Glycerophospholipid metabolism
	LysoPC (16:0)	0.71	0.02	1.35	down	Glycerophospholipid metabolism
	LysoPC (18:3 (9*Z*, 12*Z*, 15*Z*))	0.71	0.02	1.35	down	Glycerophospholipid metabolism
	Xylulose 5-phosphate	1.36	0.02	1.42	up	Pentose phosphate pathway
	d-Ribulose 5-phosphate	1.36	0.02	1.42	up	Pentose phosphate pathway
	Ribose 1-phosphate	1.36	0.02	1.42	up	Pentose phosphate pathway
	d-Ribose 5-phosphate	1.36	0.02	1.42	up	Pentose phosphate pathway
NEG	LysoPC (20:4 (8*Z*, 11*Z*, 14*Z*, 17*Z*))	0.59	0.03	1.45	down	Glycerophospholipid metabolism
	LysoPC (20:4 (5*Z*, 8*Z*, 11*Z*, 14*Z*))	0.47	0.034	1.78	down	Glycerophospholipid metabolism
	PE (14:1 (9*Z*)/14:1 (9*Z*))	0.10	0.02	2.92	down	Glycerophospholipid metabolism
	Galactosylglycerol	2.28	0.01	1.85	up	Glycerolipid metabolism
	Eicosapentaenoic acid	1.49	0.03	1.28	up	Biosynthesis of unsaturated fatty acids
	6-Phosphogluconic acid	1.78	0.02	1.38	up	Pentose phosphate pathway
	Adenosine monophosphate	1.59	0.03	1.30	up	Regulation of lipolysis in adipocytes

FC: fold change; VIP: importance of variable.

## Data Availability

These data are contained in the article.
